# Public-non-governmental organisation partnerships for health: an exploratory study with case studies from recent Ghanaian experience

**DOI:** 10.1186/s12889-016-3636-2

**Published:** 2016-09-13

**Authors:** Martin Hushie

**Affiliations:** Department of Behavioural Sciences, School of Allied Health Sciences, University for Development Studies, P.O. Box 1883, Tamale, N/R Ghana

**Keywords:** Public-non-governmental organisation partnerships, Ghana, Health reforms, Health sector, Global health, Qualitative research

## Abstract

**Background:**

The last few decades have seen a dramatic increase in public-non-governmental organisation (NGO) partnerships in the health sector of many low- and middle- income countries (LMICs) as a means of improving the public’s health. However, little research has focused to date on the nature, facilitators and barriers of these partnerships.

**Methods:**

In-depth qualitative interviews were conducted with 17 participants from five different NGOs and their collaboration with state partners in the Ghanaian health sector at the national and local levels in four regions of the country (Northern, Upper East, Greater Accra, and Eastern) to explore the drivers and nature of these partnerships and their advantages and disadvantages in the effort to improve the public’s health.

**Results:**

Major findings reveal that: 1) each collaboration between civil society organisations (CSOs) and the state in the health sector demands different partnerships; 2) partnership types can range from equal, formal contractual, decentralized to advocacy ones; 3) commitment by the state and NGOs to work in collaboration lead to improved service delivery, reduced health inequities and disparities; 4) added value of NGOs lies in their knowledge, expertise, community legitimacy, ability to attract donor funding and implementation capacity to address health needs in geographical areas or communities where the government does not reach and for services, which it does not provide and 5) success factors and challenges to be considered, moving forward to promote such partnerships in other LMICs.

**Conclusions:**

Recommendations are offered for NGOs, governments, donors, and future research including studying the organisational effectiveness and sustainability of these partnerships to deliver effective and efficient health outcomes to recommend universal best practices in health care.

**Electronic supplementary material:**

The online version of this article (doi:10.1186/s12889-016-3636-2) contains supplementary material, which is available to authorized users.

## Background

The past few decades have seen a growing number of public-non-governmental organisation (NGO) partnerships in the health sector of many low- and middle- income countries (LMICs), which include Ghana, Pakistan, Bangladesh Malawi, Tanzania, and Uganda as a means of improving the public’s health [1–5]. The implementation of various economic liberalisation and market-based health reforms, particularly from the 1980s onwards to improve health systems’ performance challenges connected to access, quality and efficiency of services, are considered significant factors contributing towards this explosion [6–9].

Increasingly, health sector reforms, postulated within a neoliberal development agenda have often resulted in dramatic changes in the institutional arrangements for the delivery of health care, and hence, the role of and the relationships between the state, civil society and private sector organisations. Nowhere is this more evident than in the context of health care in LMICs, where NGOs have been assuming a rapidly expanding role with the decline of the state as the dominant actor in the delivery of public sector functions and services [10–13].

While these new modes of organising and delivering health care have become increasingly common in many LMICs based on the belief that they enhance the effectiveness and efficiency of public health services delivery, there has been limited research concerning their derivation, constituent elements, variety, benefits or drawbacks. Moreover, there is little guidance for those interested in leveraging such partnerships at the policy and practice level. This dearth of knowledge is due in part to the lack of a clear evidence base that describes the nature and consequences of public-NGO partnerships in the health sector. This study was therefore designed to address this gap in the research on such partnerships to increase our understanding of how best NGOs might be successfully engaged as partners in the delivery of more equitable and effective health care in LMICs.

### Ghana’s health policies and systems’ challenges

In Ghana, the government adopted the Primary Health Care (PHC) strategy of 1974 as the means for achieving “Health for All” by the year 2000. The economic recession of the early 1980s radically reduced resource allocation to the health sector, resulting in a worsening of the public’s health. Ghana has since 1997, been implementing 5-year Medium-Term Health Strategies (MTHSs) that provide frameworks for health development, based on the principles of decentralization and integration in service delivery and management [14–16]. Moreover, the government’s wider development agenda in the last few years has focused on transforming Ghana into a middle income country by the year 2015, through various policies, such as the Ghana Poverty Reduction Strategies (2003–2005; 2006–2009); Millennium Development Goals (MDGs) and Ghana Shared Growth and Development Agenda, 2010–2013. The major strategic objectives of these policies over the years have included the need to: 1) increase access to quality health services; 2) improve governance and strengthen efficiency and effectiveness in health service delivery; 3) bridge equity gaps in access to health care services; 4) ensure sustainable financing arrangements that protect the poor 5) and, 6) foster collaborations to improve health with the ultimate goal of re-allocating health resources, especially, to the needy and deprived communities [17–20] - all of which have resulted in significant improvements in the wider population’s health status [21, 22]. However, gross disparities still prevail in accessibility and utilization of quality health care, which benefits the southern and urban areas of the country to the detriment of the Northern and rural areas. Consequently, parasitic and infectious diseases, malnutrition, poor reproductive health and a recent surge in non-communicable diseases remain the bane of the majority of people, especially, rural dwellers [23].

The challenge for Ghana is how to address these issues in the health sector while ensuring public, private-for-profit and non-profit provision is not compromised amidst increasing financial constraints. In this context, the government’s health policy since 1997 has increasingly exhorted and validated a change in government policy relating to organisational relationships within the health sector, in which fostering “partnerships” and collaborations” with NGOs and civil society in service provision has been recognised as means of achieving national health goals.

To date, there is evidence that the government collaborates with some Faith-Based Organisations (FBOs) and NGOs in the health sector, but much is still unknown about the drivers and nature of such partnerships and their positive and negative consequences. How governments and NGOs become ‘joined- up’, generate collective goals and deepen their collaboration; or how their interface limit and shape the search for effective organisational arrangements in the health field, is however, poorly understood. This study’s aim was to provide an investigation of the processes and elements of these partnerships and their advantages and disadvantages. The specific research questions that were qualitatively explored are as follows: 1) how do public-NGO partnerships emerge?; 2) what are the elements that constitute these partnerships?; 3) what types of partnerships can be distinguished in the health sector? and 4) what are the advantages and disadvantages of these different partnership types?

This study makes a contribution by providing a clearer understanding of on-going state-NGO partnerships among public health policy makers and implementers and informs current debates about how such partnerships can best be leveraged to achieve Universal Health Coverage around the world.

### Conceptually locating NGOs, public in partnership

In the effort to analyse the increasing engagement of NGOs as state partners in the health sector, it is important to provide operational definitions of two key terms within the institutional and organisational contexts in which these new modes of organising are emerging which are: the “public” sector and “NGO”. The “public” sector can be defined as that part of a country’s economy, which is controlled or supported financially by the government. It comprises the administrative executive branches of the state that include key ministries and agencies at the centre of government and other decentralised sector ministries and agencies that provide services such as health, education and other services with funding, policy direction and oversight from government [24]. In this research, such public institutions or organisations were conceptualised to include, the Ministry of Health (MOH), Ghana Health Service (GHS) and the Ghana AIDS Commission (GAC) with whom NGOs might collaborate to pursue common health care objectives.

The term NGOs can be used to describe all voluntary, non-profit, non-state social organisations, with varying functions; levels of operation; organisational structures; goals and membership. They include citizens’ groups or associations providing social services (e.g. health and education); development-oriented organisations; lobbying and advocacy groups seeking to effect changes in public policies that adversely affect the poor and marginalised in society [25]. NGOs form part of a broader civil society used to classify persons, institutions, and organisations such as trade unions, professional associations, church organisations, business and other special interest associations and the media [26].

## Methods

### Study design

This study can be characterised as exploratory and qualitative building on multiple case studies.

Case studies are particularly useful where the purpose of the study is to provide a holistic understanding of how and why certain events or decisions have occurred in their real contexts over time, and for studying health systems, which tend to be characterised by rapidly changing environments [27–30].

### Sampling

Following the research questions, this study aimed to obtain a theoretically relevant sample, rather than one which exactly represents the empirical population of ‘public-NGO partnerships’ at a particular time in this country. Thus, the purpose was for the sample to have representation of different NGOs and their collaborations with any state institution in the health sector so as to capture the theoretical breadth of these partnerships. Accordingly, a combination of convenience sampling: recruiting accessible organisations/participants; and snowball sampling with recruited participants suggesting other participants/organisations were used to select different NGO and government participants [31]. Given that an official register of these partnerships did not exist, it was determined that identified partnerships should at best represent four regions in Ghana: Northern and Upper East in the Northern Sector, Eastern and Greater Accra in the Southern Sector to reflect an existing North-south/rural-urban divide in geographical access to health care resources in which the Northern Sector and rural areas tend to be the most disadvantaged. Subsequently, internet searches were done to identify potential NGO participants who were then contacted, briefed on the study to find out if they were currently or had previously been in collaboration with any state institution in the health sector. Once confirmed, their willingness to participate was sought to interview a representative of the partnership who in turn suggested other participants. Eventually, eleven (11) NGOs with previous or existing collaboration with state partners were identified but only five (5) participated. The remaining six could not be included because either the NGO or government representative of the partnerships was not available at the time of the interviews.

### Data collection

Multiple sources of data were utilised and triangulated to gather data within each case study and across cases to enhance the reliability and external validity of the findings in this study [29]. The data sources were comprised of semi-structured interviews (Additional file 1) and internal documents of the government and NGOs, NGOs’ literature in health care and internet information - all of which were used to provide corroborating or contradictory evidence about the partnerships. In all, 17 semi-structured interviews were conducted in the 4 selected regions of the country (Northern, Upper East, Greater Accra and Eastern) in 2012: (11) with frontline public sector and NGO participants at the local level who worked in capacities such as Focal Person, Field or Programme Officer or Coordinator and (6) others who worked at the national levels of the partnerships in roles such as Executive Secretary or Director; Programmes or Projects Director or Officer.

### Data analysis

The interviews were digitally voice-recorded in English and transcribed verbatim. The analysis of this considerable volume of data followed a systematic approach to the study of transcripts which involved the tentative deduction of central concepts and constructs for subsequent inductive theory development within and across the case studies [32]. This approach requires that codes are developed to provide a basis for categorising and analysing the raw data on each partnership i.e., interviews and documentation [32] along five themes that were relevant for the key research questions of the study: (i) drivers of the partnerships; (ii) the contribution of partners; (iii) administration; and (iv) advantages; and (v) disadvantages of the partnerships. This was a highly iterative procedure that involved moving back and forth between the raw data collected for each partnership (i.e., interviews and documentation) [33, 34]. The final stage of the analysis entailed a cross-site analysis to identify common themes emerging across the different case studies.

## Results

### Five case studies of public-NGO partnerships

The reported growing engagement of NGOs in Ghana’s health sector has led to far-reaching changes in the institutional and organisational arrangements for health services delivery. This section reports on fieldwork of five case-study partnerships with distinct modes of NGO involvement, which are, the Catholic Relief Services (CRS) - GHS (to reduce under-five and maternal mortality); GAC – West Africa AIDS Foundation (WAAF) to combat Human Immunodefiency Virus/Acquired Immune Deficiency Syndrome (HIV/AIDS; Alliance for Reproductive Health Rights (ARHR)-GHS (to increase access to women’s sexual and reproductive health services); GHS-Sightsavers (to increase access to eye care services); and BasicNeeds- GHS (to increase access to mental health services). Each case is described using the five themes that guided this study: (i) partnership drivers; (ii) partners’ contribution; (iii) partnership administration; (iv) advantages and (v) disadvantages summarised in Tables 1, 2, 3, 4 and 5. In presenting findings, the anonymity of participants is preserved by only referring to the names of the organisations they represent.

**Table 1:** Summary findings: CRS- GHS partnership

Region of location	Upper East (Talensi- Nabdam and Kassena-Nankana West Districts)	Form of involvement (initiation, contribution of partners and management of the collaboration)	Advantages	Disadvantages
Interviews conducted	2 NGO 2GHS	- Dubbed “CIMACS” - Initiated by GHS with CRS as an expert partner/and funder. - An MOU signed. - GHS supported with health facilities and personnel. - CRS trained Traditional Birth Attendants (TBAs) and community volunteers in social mobilization skills, provided logistics/incentives to attract pregnant women to deliver in health centres. - Program officers of CRS and focal persons of GHS shared equally in program management and regularly exchanged project progress information. - GHS held review meetings at sub-district, district and regional; quarterly, half yearly and annual basis, to evaluate progress with CRS/stakeholders. - CRS maintained monitoring and evaluation systems e.g. conduct of baseline studies; mid- term and end-of-project evaluations. - Quarterly and annual reports generated and shared with GHS/ donors as a requirement for continued support. - GHS required to keep records of CRS support (material and cash) and to render accounts periodically.	Improvement in maternal and child health indicators-e.g. antenatal registration (25 %); antenatal clinic attendance (22 %); exclusive breastfeeding (25 %); institutional deliveries (55 %); [35] and increased drug and immunization uptake.	- Occasional tendency for some GHS staff to provide somewhat unreliable data not useful for effective programming; - Occasional non-disclosure of full budget lines by GHS - Lack of synchronization of each other’s timetables; - CRS’ eagerness about timelines to demonstrate program performance to donors.
Driver of Collaboration	High under-five and maternal mortality recognized by GHS; - Need to attract pregnant women to deliver in government health centres rather than with TBAs.			
Time Frame	2009–2011

**Table 2:** Summary findings: GAC- WAAF partnership

Region of location	Greater Accra	Form of involvement (initiation, contribution of partners and management of the collaboration)	Advantages	Disadvantages
Interviews conducted	1 NGO 1 GAC	- Initiated by GAC through contract agreement with WAAF (NGO). - WAAF engaged in information, education and communication materials distribution; condom distribution/HIV testing and counseling services. - GAC coordinated and disbursed external donor funding from World Bank, Department for International Development, United Kingdom (DFID, UK) and Danish International Development Agency (DANIDA) through ‘large’ CSOs believably with better organizational capacities for disbursement to ‘smaller’ community-based organisations (CBOs), NGOs, and FBOs to implement programs at the local/community levels to build CSOs’ capacities for service delivery. - GAC maintained a projects/technical/ monitoring and evaluation (M & E) and finance divisions for effective program coordination. - Regular meetings held for CSO implementers to exchange information exchange, share knowledge and appraise performance. - Implementation results often collated into a database and reported on to stakeholders, including donors. - GAC’s internal audit and technical departments ensure proper application of funds.	Built capacity and credibility of NGOs for HIV/AIDS service delivery to reduce the epidemic’s spread.	- Occasional delays in release of donor funds by GAC; - high staff turnover rate among NGOs.
Driver of Collaboration	High HIV/AIDS prevalence			
Time Frame	2005–2006

**Table 3:** Summary findings: ARHR- GHS partnership

Region of location	Upper East	Form of involvement (initiation, contribution of partners and management of the collaboration)	Advantages	Disadvantages
Interviews conducted	2 NGO 2 GHS	- Initiated by ARHR with funding from CORDAID and formalized through signing of an MOU with GHS; - GHS contributed technical expertise and existing health facilities. - ARHR mobilized and educated project beneficiaries on their rights to demand and use existing state-provided services; - ARHR had the GHS to organize district, sub-district and community- level interface meetings with various stakeholders, including the district assemblies to provide opportunity for community members to share their concerns regarding existing health care services and how to address them. Action plans, addressing issues were drawn and implemented with ARHR support. - Projects evaluated through community feedback meetings for sharing information and project implementations issues. - ARHR periodically engaged external assessors to do participatory evaluation with GHS, community members and other stakeholders.	- GHS recognized ARHR’s role as “gap filler” making-up for government deficits in health care through its resource mobilization capacities, innovative ideas, expertise, and timeliness in the delivery of programs. - Effective reach of target populations with maternal and child health services (family planning, antenatal care and skilled delivery). - Strengthening of ARHR’s advocacy and rights-based approaches to improving women’s health.	- Delays caused by long government bureaucratic procedures required to implement programs. - Occasional tendency for some GHS staff to provide relatively unreliable data hardly useful for effective programming. - Health systems’ challenges (e.g. shortage and inequities in the distribution of human resources and ill-equipped health facilities.
Driver of Collaboration	- Baseline study indicated inadequate access to women’s sexual and reproductive health services.			
Time Frame	2007–2010

**Table 4:** Summary findings: Sightsavers- GHS partnership

Region of location	Eastern	Form of involvement (initiation, contribution of partners and management of the collaboration)	Advantages	Disadvantages
Interviews conducted	1NGO 2 GHS	Initiated by GHS with external funding from Sightsavers with periodic signing of an MOU. - GHS supports with government eye clinics, equipment, consumables and human resources. - Sightsavers provides financial and technical support for implementing programs and projects, including infrastructure provision (eye clinics); human resource development: training of ophthalmologists and eye care personnel; and facilitation and importation of essential drug donations and equipment. - Regional Health Directorate of GHS hosts the partnership’s secretariat managed by a Program Manager. This secretariat has a technical wing, made up doctors, nurses and district health institutions that provide services. The Regional Director of Health Services (RDHS) heads the secretariat, assisted by the Deputy Regional Director of Health Services and the Regional Eye Care Coordinator. - The district health institutions channel needed logistical supplies and resources through the Eye Coordinator to the RDHS for submission to Sightsavers and vice- versa from Sightsavers to the institutions. - GHS holds stakeholders’ meetings regularly (e.g., mid-year, end-of-year) for Sightsavers, government and other stakeholders, to identify program priorities, costs and implementation strategies, and to evaluate progress.	- Reduced burden of eye diseases estimated at a relatively lower prevalence: 0.7 % or 18400 of this region’s population (2,633,154) contrasted with national level prevalence: 1 % or 240,000 of Ghana’s population (24 million) [36] - Increased number of trained ophthalmologists (from 2 to 4); ophthalmic nurses (from 12 to 32); optometrists (2 to 8); equipment technicians (1 to 5) and, eye centres (from 10 to 18) providing 24- h services – Availability of basic equipment (e.g. slit lamps, torch lights and ophthalmoscopes, vehicles for outreach and primary eye care services.	- High staff turnover among Sightsavers’ employees - GHS’ inability to keep program/project timelines or deadlines
Driver of Collaboration	High burden of eye diseases			
Time Frame	1996 to date

**Table 5:** Summary findings: BasicNeeds- GHS partnership

Partnership name	BasicNeeds-GHS	Form of involvement (initiation, contribution of partners and management of the collaboration)	Advantages	Disadvantages
Region of location	Northern	- Initiated by BasicNeeds in 2002 with formal MOU signed in 2009. - BasicNeeds provides funding from external donors and international NGOs such as European Union – Brussels, DFID, African Women Development Fund and Comic Relief, UK for supporting: 1) psychiatric nurses’ allowances; 2) fuelling of GHS vehicles for outreach services; 3) procurement of psychotropic medicines; 4) cost of hiring psychiatrists from the southern part of the country for outreach services in the north; 5) training of community-based volunteers in disease surveillance, basic symptom recognition and case search; 6) livelihood activities of the mentally-ill and their families and 7) empirical research evidence for its advocacy work and efforts to integrate traditional healers, users of services, and caregivers of the mentally- ill into the national PHC system. - GHS supports with existing government health facilities (hospitals/health centres/clinics/psychiatric units), personnel, vehicles and occasional funding. - As a mental health advocate, BasicNeeds achieves its objectives through relations that are sought and established with various state organizations such as, the MOH, GHS at the national level, while working with community psychiatric nurses in health centres and district hospital at the PHC level. - Partners hold periodic review meetings to evaluate performance and promote continuous self- improvement. - BasicNeeds shares information such as research findings on mental health issues, brochures and picture books that show images and the conditions of the mentally- ill, with government partners and the public through the electronic and print media to create awareness of this country’s mental health situation.	- Increased government awareness of mental illness and mental health issues; - Increased government support for mental health care: infrastructure and human resources (trained psychiatric doctors and nurses, clinical psychologists and social workers; - Awareness creation and promotion of societal interest in the treatment and stabilization of the mentally-ill - Source of credibility and legitimacy for BasicNeeds	- Delays associated with cumbersome government bureaucracy in procuring psychotropic medicines from GHS; - GHS’ slow progress made to date in integrating mental health services into PHC services.
Interviews conducted	2 NGO 2 GHS			
Driver of Collaboration	Baseline study revealed high burden of mental illness/ Epilepsy; - inadequate access to government mental health services.			
Time Frame	2002-to date			

### CRS-GHS partnership

The CRS-GHS partnership (Table 1), dubbed “Community Initiative on Maternal, Child and Newborn Survival” (CIMACS), was initiated by GHS in the Upper East Region of Ghana (i.e. Talensi-Nabdam and Kassena-Nankana West Districts) between 2009 and 2011, to reduce an increasing burden of maternal, child and neonatal deaths, attributed to pregnant women’s preference to deliver with Traditional Birth Attendants (TBAs) rather than government skilled birth attendants. Following CRS’ identification as a potential partner, the parties signed a Memorandum of Understanding (MOU) to commence the project.

### GAC-WAAF partnership

The GAC- WAAF partnership (Table 2) started following GAC’s establishment in 2002, as the coordinating agency in Ghana’s HIV/AIDS response, and its subsequent implementation of the World Bank’s Multisectoral HIV/AIDS Programme in 2005 to fight the disease through treatment, prevention, care and support strategies. Under this programme, CSOS/NGOs needed to apply to be engaged as service providers. WAAF was one such NGO contracted as GAC’s implementing partner.

### ARHR-GHS partnership

The ARHR-GHS partnership (Table 3) initiated by the former in the Bongo district of the Upper East Region of Ghana, was funded by the Catholic Organisation for Relief and Development Aid (CORDAID) and implemented between 2007 and 2010 with a view to: 1) achieve the health-related MDGs through capacity building for research and evidence-based advocacy; 2) educate and instil a sense of right among community members to demand services and 3) promote participatory monitoring and evaluation and accountability in service delivery. The main driver was an ARHR-led baseline study which identified this district as lagging behind in achieving the health-related MDGs. A negotiation and signing of an MOU with the MOH/GHS followed this to commence the partnership.

### GHS - Sightsavers partnership

The GHS- Sightsavers collaboration (Table 4) started in the Eastern Region of Ghana in 1996, when an increasing burden of eye diseases, including Onchocerchiasis, blindness from cataract and other eye conditions prompted the GHS to engage Sightsavers to help, for which an MOU, renewable every 5-years was endorsed. Initially, Sightsavers operated as a charity, funding the government’s identified eye care needs. However, since 2005/2006, its operational strategy changed to seeking an integration of eye care services into the national, regional and district PHC system including the government’s annual work plans and budgets. The purpose has been to promote state ownership and institutionalisation of services, following the World Health Organisation’s (WHO’s) Vision 2020 agenda for strengthening LMICs’ health systems.

### BasicNeeds - GHS partnership

The BasicNeeds-GHS partnership (Table 5) was initiated in Northern Ghana by the former in 2002, following a baseline study, which revealed that existing government psychiatric units when compared with facilities in this country’s southern sector lacked the requisite personnel and logistics for handling an increasing burden of mental illness and epilepsy. Subsequently, BasicNeeds sought to increase access to services for the mentally-ill with GHS until 2009, when an MOU was agreed.

## Discussion

This study has provided an overview of the emergence and flourishing of various forms of public-NGO partnerships in Ghana’s health sector, and the increasingly important role these new governance arrangements are playing towards achieving wider health systems’ goals. While these five cases represent different forms of NGO involvement in the health sector, they cannot be considered representative of the range of such partnerships, nor as best practices. Nonetheless, they offer a window into the world of state-civil society partnerships throughout the country, and by extension in the health sectors of LMICs that are working to improve community health, reduce disparities, promote equity and strengthen the health system.

In particular, the findings reveal that each collaboration between the state and civil society in the health sector demands different partnerships. A partnership form and function reflects the need to address complex and diverse global health-related needs that presumably go beyond the capacity of either the state or NGO to effectively tackle alone. These were in the fields of maternal, newborn and child health; HIV/AIDS treatment and prevention; women’s sexual and reproductive health; eye care and mental health services. Each of the collaborations was thus context-specific, facilitated through different processes, was often engaged with different objectives, adopted different management structures and made varying contributions towards improving the public’s health.

Secondly, this study tells of the added value of NGOs in partnerships. This includes their knowledge/innovative ideas, expertise, community mobilisation and empowering skills, social legitimacy, implementation capacity and ability to attract external donor funding to address diverse health needs in under-served or un-served geographical areas or communities and for services that the government did not provide or were least prioritised in its public health agenda.

Moreover, it is possible to generalise these different forms of NGO involvement into at least four major inter-related categories as “equal”, “formal contractual”, and “decentralised” and “advocacy” partnerships, by their nature and functioning. Thus, the CRS-GHS collaboration can be characterised as an equal partnership, regarding how it revolved mostly around shared objectives, equitable responsibilities and decision-making processes. The GAC-WAAF partnership can be described as formal contracting one by its results-based character, in which the former explicitly specified a series of objectives and indicators by which to measure the latter’s performance; and efficiency focus, using formalised administrative structures and procedures such as the collection of data to demonstrate programme effectiveness as a condition for reward and continued engagement. Moreover, the GHS-Sightsavers partnership may be considered as a decentralised one, in relation to how the GHS hosts the partnership’s secretariat, acting as the lead partner and holding ownership and responsibility for the management of the partnership. Finally, the ARHR-GHS and BasicNeeds-GHS partnerships can be branded as advocacy ones, as the NGO partners championed ideas, strategies and methods to increase the availability and accessibility of state-provided women’s reproductive and mental health services for the poor and deprived in society.

### Success factors across the partnerships

Many of the factors contributing to the success of the case studies in this research have been conceived as context-specific and unique to a particular project and partnership. Nonetheless, several factors can be inferred as facilitators of effective functioning and outcomes from a cross-case analysis that can serve as a guide for replicating such partnerships in other LMICs. These include: 1) developing new relationships by adopting and implementing health needs-based approaches and evidence-based interventions; 2) commitment to mobilise internal and external resources and support for effective programming; 3) using MOUs to formalise expectations for collaborative relationships as well as respective project roles and responsibilities; 4) making programme planning and implementation a collaborative process by involving project partners and key stakeholders from start-up to the end; 5) ensuring monitoring and evaluation is a continuous process to identify programme needs and issues and to engage in continuous programme improvement; 6) using decentralised organisational and administrative structures and existing country systems to promote local ownership and sustainability of programmes; and 7) sharing of accurate and timely information among partners, stakeholders, donors and the public to ensure more effective programme outcomes.

### Challenges of the partnerships

This study has attended to several challenges that may be viewed as grounded in the contexts, structural and interpersonal realities surrounding a particular health project or programme and public-NGO partnership working. These ranged from the tendency of some GHS officials to provide seemingly suspicious data hardly useful for effective programming (CRS-GHS) to GAC’s occasional delays in releasing donor funds (GAC-WAAF) and the GHS’s limited progress made to integrate mental health services into the national PHC system (BasicNeeds-GHS). Nonetheless, the partnerships also faced several common challenges: difficulty of synchronising each other’s work programmes; high staff turnover rate among CSOs; lack of transparency and mutual suspicion among partners’ financial contributions, delays caused by cumbersome government bureaucratic procedures; government’s inability to keep set programme targets, timelines or deadlines; NGOs’ eagerness about timelines to demonstrate programme effectiveness to donors; and broader health systems’ challenges, such as shortage and inequities in the distribution of human resources and ill-equipped health facilities: challenges which can be managed through targeted new polices and interventions moving forward to more effectively promote these partnerships in other settings.

### Limitations and future research

This study suffers from limitations. First, despite the attention paid to the selection of the case-studies, the analyses remain exploratory and not meant to be conclusive, focusing on the factors associated with the successes and challenges of these partnerships rather than addressing their long-term impact. Future studies within a longitudinal framework are needed to examine the organisational effectiveness and sustainability of these partnerships in delivering effective and efficient health outcomes. Second, this study’s sample is purposive and non-random, based on a small sample of these partnerships at a single point in time: the accuracy of generalising to other countries cannot be guaranteed. However, as was highlighted in the methodology section, the study’s emphasis on a theoretically valid sample implies that the explored concepts and relationships about public-NGO health partnerships may be validly examined in other contexts. Finally, since this study focused mainly on public-NGO (non-profit) partnerships as a distinct mode of organising, the findings cannot be assumed to apply to other types of collaborations such as public- private-for-profit (business) partnerships, which may exhibit differences in their formation, nature and consequences. Replicating this study among other partnership types may be necessary in order to strengthen its theoretical generalisability.

## Conclusions

In order to optimise the future impact of these partnerships based on the findings presented here, related recommendations are made for health NGOs, governments and donors. For health NGOs, this study attends to how they can take initiative to gain access to combinations of resources from internal and external sources to develop innovative policies and approaches to addressing global health challenges, maintain legitimacy and survive. Moreover, dynamic new partnerships can offer NGOs better channels of engagement with the wider community and greater capacity to influence the health policy agenda.

Moreover, the findings here demonstrate that the government recognises the contribution of NGOs to the health field. However, on-going interactions appear to be ad hoc and voluntary in nature to suggest lack of strategic approaches for engaging NGOs in the health sector. Nonetheless, given the intractable challenges governments in many LMICs face in health care finance, management and provision, this study underscores how they can design and implement new innovative partnerships with NGOs through specific policies, programmes and strategies to harness their individual strengths, resources and expertise in a complementary fashion to improve the public’s health.

Additionally, this study has emphasised how these partnerships would tend to be limited to projects funded through external donor assistance that is mobilised by NGOs. However, in an era of dwindling external donor support, the future role of NGOs in these partnerships will depend largely on their ability to obtain funding from within, and governments in LMICs have a crucial role to play in accelerating the process of developing effective mechanisms for generating such resources for health development. Finally, this study underscores features that characterise effective and sustainable government-NGO partnerships, which can serve as a useful resource for donors, and international NGOs interested in expanding health care services in the developing world.

## Additional file

Additional file 1: Interview Guide for Case Partnership Representatives. (DOCX 24 kb)

## Abbreviations

ARHR: Alliance for Reproductive Health Rights
CBO: Community-Based Organisation
CORDAID: Catholic Organisation for Relief and Development Aid
CRS: Catholic Relief Services
CSOs: Civil society organisations
DFID: Department For International Development, UK
FBOs: Faith-Based Organisations
GAC: Ghana AIDS Commission
GHS: Ghana Health Service
HIV/AIDS: Human Immunodefiency Virus/Acquired Immune Deficiency Syndrome
LMICs: Low and middle income countries
M&E: Monitoring and evaluation
MDGs: Millennium Development Goals
MOH: Ministry of Health
MOU: Memorandum of Understanding
MTHSs: Medium-Term Health Strategies
NGO: Non-governmental organisation
Organisation CIMACS: Community Initiative on Maternal, Child and Newborn Survival
PHC: Primary Health Care
TBAs: Traditional Birth Attendants
WAAF: West Africa AIDS Foundation
WHO: World Health Organisation
