# Supplementary material for: Public-non-governmental organisation partnerships for health: an exploratory study with case studies from recent Ghanaian experience
Source: BMC Public Health. 2016 Sep 13;16(1):963. doi: 10.1186/s12889-016-3636-2 (PMC5020518; doi:10.1186/s12889-016-3636-2)
Supplement: Additional file 1: — Interview Guide for Case Partnership Representatives. (DOCX 24 kb) [file 12889_2016_3636_MOESM1_ESM.docx]

Interviewer's initials: _________ Today's date: __________

Start time: ____________

**Interview Guide for Case Study Partnership Representatives**

Public-Non-Governmental Organisational Partnerships for Health in Ghana

Overview: The intent of this interview guide is to gather information on Public-Non-Governmental Organisational Partnerships for Health in Ghana. This document is intended to guide interviewers through the key content areas of data collection for this project, ensuring that the same content is discussed with each participant. While the interview guide is used to facilitate the discussion, it is not a rigid script that must be adhered to verbatim. This ensures that the interviewer gathers data on the same topics with each individual, while also allowing the flexibility to adapt and clarify questions to suit the needs of different participants. Similarly, questions need not be asked in this particular order. The facilitator will adapt the conversation for each person, pursuing both the *a priori* research topics as well as any emergent relevant themes that evolve during the interview.

**Welcome procedures**

*Interviewer: Welcome participant, explain basic study details and purpose, explain that interviews are being recorded, obtain informed consent, and discuss how we will keep this information private, anonymous, and confidential.*

**General Content Areas**

1. **General Information**
   1. How would you characterize your organization (e.g., civil-society organization, non-governmental organization, community-based organization, faith-based organization, or other)?
   2. What is your current title or position at your organization?
   3. How long have you been at this organization?
2. **Drivers of the partnership**
   1. How did the idea of the partnership start? (Describe in detail the processes involved in how the partnership started).
   2. What motivated your organization to engage in the partnership?
   3. Did your organization have other options than engaging in this partnership, and if so what were they?
   4. What did you see as some of the advantages/benefits to be gained by your organization in collaborating with your partner?
   5. What did you see as some of the disadvantages/risks for your organization by going into the partnership?
   6. What other factors/issues did you consider when considering entering into the partnership?
   7. How did your organization eventually settle for the partnership?
   8. What did you consider when deciding which organization to include in the partnership?
   9. Did you consciously decide to include/exclude any particular organizations or types of organizations?
   10. What factors did you consider when considering what form the partnership should take?
   11. What shared values brought the two of you together?
3. **Nature of the partnership**
   1. **Purpose**
   2. What are the current goals of the partnership?
   3. To what extent are the goals of your organization similar to the goals of your partner organization?
   4. Which aspect of health care are you into in the partnership?
   5. Who are the target populations?
   6. What specific solutions does your organization provide for the partnership?
   7. To what extent would you suggest that your partner organization relies on a mission statement for the collaboration different from yours?
   8. **Governance/management**
4. Briefly describe the relationship between your organization and partner.
5. How is the partnership managed administratively?
6. To what extent do you rely on a formal agreement that spells out relationships between partner organizations?
7. To what degree do you rely on standard operating procedures (like rules, policies, forms) created by partner organizations to coordinate each other’s activities in the collaboration?
8. Do you have a board or steering committee specifically created for making decisions about the collaboration? If so, how are decisions made about the goals and activities of the collaboration, and how often does your organization participate in such decision-making processes?
9. To what extent does your partner organization take your organization’s opinions seriously when decisions are made about the collaboration?
10. Under what circumstances do you rely on informal personal relationships or procedures with your partner organization when making decisions about the collaboration?
11. Do you sometimes feel your partner organization is keeping an eye on your organization’s activities to make sure you are doing what you are supposed to be doing in the collaboration? If so, how do you know?
12. Does your organization keep an eye on partner organizations’ activities in the collaboration to make sure they are doing what they are supposed to be doing in the collaboration? If so, how is this done?
13. How do you handle problems facing the collaboration?
14. What mechanisms do you have in place for evaluating outcomes of the partnership to ensure that you learn from each other’s experiences?
15. What different types of information and knowledge do you share with your partner organization?
16. What mechanisms or channels are in place for sharing such information and knowledge across the partnership? (What are the means by which you share relevant information, experiences or knowledge with each other in the partnership?
17. Does your organization have problems getting in touch with partner organizations when you need to contact them? If so, how are such problems resolved?
18. What information is your organization willing to share with partner organizations for the good of the collaboration even though you would be better off withholding it?
    1. **Resources**

1. What different types of resources does your organization (e.g. financial, expertise, time, legitimacy, reputation etc) bring to the partnership?

2. To what extent would you suggest that your organization needs the resources, services, or support of the partner organization to accomplish its goals?

3. To what extent would you suggest that your partner organization need the resources, services, or support of your organization to accomplish their goals?

- 1. **Trust**

1. What mechanisms do you have in place to ensure that the partnership operates in an open, credible and fair manner?
2. In what situation(s) have you found your partner organization taking advantage of the collaboration because your organization was found vulnerable in a way?
3. How far can you count on your partner organization to meet its obligations to the collaboration?
4. Have you had occasions where your partner organizations try to get the upper hand when they negotiate in the collaboration? Can you cite any such occasion?
5. Have you at some time during the partnership felt like leaving because your organization has been treated unfairly?
6. Does your organization sometimes feel like pursuing its own interests even at the expense of the partner organization? Can you cite some of those circumstances?
7. Under what conditions do you see your organization as having a duty to meet its obligations to its partner organization even if they do not always meet their obligations to you?
8. How does your organization protect its own organizational integrity in matters concerning the collaboration?
9. In what ways would you suggest that developing long-term personal relationships with partner organizations is the most important part of collaborating?
   1. **Facilitators/Advantages of the partnership**
10. In what ways has your partner organization positively influenced your organization’s services or operations?
11. How have you combined and used each other’s resources so all partners benefit from collaborating?
12. To what extent would you suggest that your partner organization appreciates and respects what your organization brings to the collaboration?
13. In what ways has your organization achieved its own goals better working with partner organizations than working alone?
14. What differences arise in the collaboration process and how do work through differences to arrive at win–win solutions?
15. Overall, what difference has the partnership made in terms of achieving its primary objectives?
16. Do you sometimes hold celebrations to recognize joint successes in the collaboration? What form do those celebrations take?
    1. **Barriers/Challenges of the partnership**
    2. What have been some of the drawbacks for your organization participating in the partnership to date?
    3. What have been some of the differences between your organization and your partner organization that makes it difficult for you to work together?
    4. What misunderstandings have you had in relation to of the issues that initially brought you to the collaboration?
    5. In what ways has the collaboration hindered your organization from meeting its own organizational mission?
    6. How has your organization’s independence been affected by having to work with partner organizations on activities related to the collaboration?
    7. In what ways have you, as a representative of your organization, felt pulled between trying to meet both your organization’s and the collaboration’s expectations?
    8. Has there been any occasion when your organization is up-front with partner organizations about what it can and cannot give (in time, money, energy, and other resources) to achieve the collaboration’s goals? Can you cite such any such occasion?
    9. How would your organization be affected if it decided to pull out of the collaboration today?
    10. What would make your organization feel it worthwhile to stay and work with partner organization rather than leave the collaboration?
    11. Within the last ten years, what do you see as some of the peculiar challenges that NGOs and government partners have faced in managing in providing health services in the country?
    12. What do you see as the way forward in effectively managing public-NGOs partnerships in Ghana’s health sector?

- 1. **Lessons of partnership**
  2. Generally, what factors would you consider as having made ongoing public-NGO partnerships in Ghana’s health sector successful?
  3. What factors would you suggest as having contributed to failures?
  4. How would you summarize the overall experience with public-NGO partnerships in the health sector?

**Closing procedures**

*Interviewer: Thank participant for their time and effort. Remind them that we will keep everything private, confidential, and anonymous. Record the time when interview was completed.*

Finish time*: ___________*
